# Urine culture–guided antibiotic prophylaxis reduces febrile pyelonephritis after ureteral stent removal following radical cystectomy

**DOI:** 10.1007/s00345-026-06334-z

**Published:** 2026-03-18

**Authors:** Hiroyuki Kitano, Eisuke Watanabe, Kayoko Tadera, Yoshinori Nakano, Shinsaku Tasaka, Kazuma Yukihiro, Mai Okazaki, Naofumi Nomura, Tomoya Hatayama, Hiroyuki Shikuma, Kyosuke Iwane, Ryo Tasaka, Yuki Kohada, Kenshiro Takemoto, Shunsuke Miyamoto, Miki Naito, Kohei Kobatake, Yohei Sekino, Keisuke Goto, Akihiro Goriki, Keisuke Hieda, Tetsutaro Hayashi, Seiya Kashiyama, Hiroki Ohge, Nobuyuki Hinata

**Affiliations:** 1 Department of Urology, Graduate School of Biomedical and Health Sciences, Hiroshima University, 1-2-3 Kasumi, Minami-ku, Hiroshima, 734-8551 Japan; 2 Section of Clinical Laboratory, Division of Clinical Support, Hiroshima University Hospital, Hiroshima, 734-8551 Japan; 3 Department of Infectious Diseases, Hiroshima University Hospital, Hiroshima, 734-8551 Japan

**Keywords:** Radical cystectomy, Ureteral stent removal, Antimicrobial prophylaxis, Urinary tract infection, Antibiotic selection

## Abstract

**Purpose:**

To evaluate whether urine culture–guided prophylactic antibiotic selection reduces infectious complications after ureteral stent removal following radical cystectomy with intestinal urinary diversion.

**Methods:**

We retrospectively analyzed 128 patients who underwent radical cystectomy with intestinal urinary diversion between February 2009 and July 2025. Patients were divided into a culture-guided prophylaxis group and an empirical prophylaxis group. The primary endpoint was febrile pyelonephritis after ureteral stent removal. Multivariable logistic regression analysis was performed to identify independent risk factors for post–stent removal infection.

**Results:**

Patients in the culture-guided group more frequently received antithrombotic therapy, neoadjuvant chemotherapy, and robot-assisted surgery, and had shorter operative times and less blood loss. Febrile pyelonephritis occurred in 2 patients (3%) in the culture-guided group and 10 patients (16%) in the empirical group (*P* < 0.05). *Enterococcus faecalis* was the most commonly isolated pathogen. Multivariable analysis identified urine culture–guided prophylactic antibiotic selection as an independent protective factor against febrile pyelonephritis (odds ratio 0.139, *P* = 0.04), while antithrombotic therapy was an independent risk factor.

**Conclusions:**

Urine culture–guided prophylactic antibiotic selection before ureteral stent removal significantly reduced febrile pyelonephritis after radical cystectomy with intestinal urinary diversion, supporting its use as an effective infection prevention strategy in this high-risk population.

**Supplementary Information:**

The online version contains supplementary material available at 10.1007/s00345-026-06334-z.

## Introduction

Radical cystectomy with pelvic lymph node dissection and urinary diversion is the standard treatment for muscle-invasive bladder cancer and is recommended in selected cases of locally advanced disease [1]. While open radical cystectomy remains the standard surgical approach, it is associated with substantial bleeding and risks of perioperative complications [2]. Laparoscopic radical cystectomy was developed as a minimally invasive alternative to mitigate these complications; however, its adoption in clinical practice has been limited due to a steep learning curve. Robot-assisted radical cystectomy, designed to overcome these technical challenges, offers advantages such as a magnified three-dimensional view and articulated robotic arms that mimic wrist-like movements, enabling more precise surgical manipulation [3]. Radical cystectomy is associated with a high postoperative complication rate. For example, in a randomized clinical trial comparing robot-assisted and open approaches, postoperative complications occurred within 90 days in 67% and 69% of patients who underwent robot-assisted radical cystectomy and open radical cystectomy, respectively. Urinary tract infection (UTI) was the most common complication, observed in 35% and 26% of patients in the robotic and open groups, respectively [4]. UTIs after urinary diversion are caused by microorganisms and may arise from bacterial colonization within the reconstructed urinary tract, particularly in bowel-derived mucosa that harbors its own microbiota. In addition, transient ureteral obstruction or edema after stent removal may facilitate ascending infection, and a second peak of infectious complications has been reported following stent removal [5–7].

This study aimed to evaluate whether prophylactic antibiotics, selected based on urine culture results obtained from the ileal conduit prior to ureteral stent removal, could reduce the incidence of UTIs after stent removal in patients who underwent radical cystectomy with ileal conduit urinary diversion.

## Patients and methods

### Study design and participants

This retrospective cohort study was approved by the Institutional Review Board of Hiroshima University (approval number: E2022-0003). The study population comprised patients who underwent radical cystectomy with ileal conduit urinary diversion or orthotopic neobladder reconstruction at Hiroshima University Hospital between February 2009 and July 2025. Other types of urinary diversion, including ureterocutaneostomy, were excluded.

Patients were categorized into two groups: a culture-guided prophylaxis group (prophylactic antibiotics selected based on pre-ureteral stent removal urine culture results) and an empirical prophylaxis group (prophylactic antibiotics determined at the attending physician’s discretion). No randomization was performed because this study was designed as a retrospective observational cohort study. Allocation to each study group reflected an institutional change in clinical practice rather than investigator-directed assignment. Specifically, around 2020, our institutional protocol was modified to introduce routine urine culture submission prior to ureteral stent removal, with prophylactic antibiotics selected based on culture results. Accordingly, patients treated before this practice change were generally categorized into the empirical prophylaxis group, whereas those treated after implementation were generally classified into the culture-guided prophylaxis group.

The primary endpoint was the incidence of acute pyelonephritis following ureteral stent removal. Pyelonephritis was clinically defined according to the European Association of Urology (EAU) guidelines on urological infections as the presence of fever (> 38 °C), chills, flank pain, nausea, vomiting, or costovertebral angle tenderness [8, 9]. The secondary endpoints were the evaluation of clinical factors associated with the development of pyelonephritis after stent removal, as well as comparisons of length of hospital stay and total hospital costs according to the presence or absence of post–stent removal pyelonephritis.

### Surgical procedure and ureteral stent management

Radical cystectomy with ileal conduit urinary diversion was performed using standard surgical techniques. Pelvic lymph node dissection was performed according to institutional guidelines and disease characteristics, and was routinely performed in all patients from October 2021 onward.

Bilateral ureteral stents were placed intraoperatively and externalized through the ileal conduit stoma, and were routinely removed between postoperative days 14 and 16.

### Urine culture and prophylactic antibiotic strategy

In the culture-guided prophylaxis group, Urine culture samples were collected from the external drainage bag connected to the ureteral stents, which had been inserted bilaterally into the renal pelvis through the ileal conduit or orthotopic neobladder. Sampling was performed between postoperative days 8 and 10 while the stents remained in situ. Prophylactic antibiotics administered at ureteral stent removal were selected based on antimicrobial susceptibility testing results, in consultation with a board-certified infectious disease specialist accredited by the Japanese Association for Infectious Diseases. Bacteria detected at a concentration of ≥ 10^2^ colony-forming units/mL were defined as target organisms for antimicrobial therapy [10].

A prophylactic antibiotic regimen encompassing all detected organisms was selected, with a standard administration duration of 48 h. When therapeutic drug monitoring was indicated, such as with glycopeptide antibiotics, administration was extended to approximately 72 h, in accordance with recommendations from a dedicated clinical pharmacist. When two prophylactic antibiotics were deemed necessary, they were administered concurrently.

In the empirical prophylaxis group, prophylactic antibiotics were administered without reference to the urine culture results, based on the attending physician’s clinical judgment.

### Data collection and outcomes

Patient demographics, perioperative clinical outcomes, and hospitalization costs were extracted from medical records and subsequently compared between the two groups.

### Statistical analysis

Group comparisons were performed using the chi-square test or Fisher’s exact test for categorical variables, and the t-test or Mann–Whitney U test for continuous variables, as appropriate. Multivariable logistic regression analyses were conducted to identify independent predictors of post-stent removal pyelonephritis. Given the limited number of events, variables were selected based on clinical relevance and biological plausibility rather than being entered indiscriminately, in order to minimize the risk of overfitting. The multivariable analysis should therefore be interpreted as exploratory and hypothesis-generating rather than confirmatory.

Multicollinearity among explanatory variables was assessed using pairwise correlation coefficients. Variables exhibiting strong multicollinearity (|r| > 0.8) were excluded from the final multivariable model, and representative variables were retained to limit overfitting. Variables indicating ischemic heart disease and cerebrovascular disease were excluded due to a strong correlation with antithrombotic therapy. Similarly, the surgical approach was excluded from the multivariable analysis because the implementation of robot-assisted radical cystectomy coincided with an institutional policy change mandating routine urine culture submission before ureteral stent removal, resulting in substantial collinearity between the surgical approach and the prophylactic strategy.

Perioperative outcomes were compared between groups using two-way repeated-measures analysis of variance with a Type III sum of squares. Statistical analyses were performed using JMP Pro version 18.1.0 (SAS Institute Inc., Cary, NC, USA) and GraphPad Prism version 10 (Dotmatics Inc., San Diego, CA, USA). All statistical tests were two-sided, and *P* values < 0.05 were considered statistically significant.

## Results

### Patient characteristics

Patient baseline characteristics are summarized in Table 1.

**Table 1:**
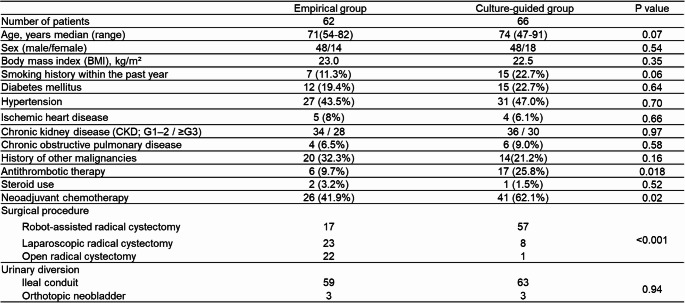
Patient characteristics (*N* = 128)

Patients in the culture-guided prophylaxis group received antithrombotic therapy more frequently than those in the empirical prophylaxis group (*P* = 0.018). A greater proportion of patients in the culture-guided prophylaxis group received neoadjuvant chemotherapy compared to the empirical prophylaxis group (*P* = 0.020). No significant differences were observed between the two groups with respect to age, sex, or other baseline demographic variables.

### Perioperative outcomes

Perioperative outcomes are summarized in Table S1.

**Fig. 1:**
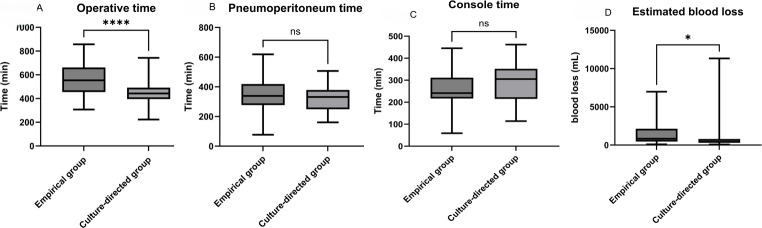
Intraoperative outcomes comparing the empirical prophylaxis and culture-guided prophylaxis groups. **A** Operative time. **B** Pneumoperitoneum time. **C** Console time. **D** Estimated blood loss

Operative time was significantly shorter in the culture-guided prophylaxis group compared to the empirical prophylaxis group (median, 395 min [range, 223–743 min] vs. 454 min [307–857 min], *P* < 0.001; Fig. 1A). No significant differences were observed between the two groups with respect to pneumoperitoneum time (331 min [160–507] vs. 339 min [181–619], *P* = 0.24; Fig. 1B) or console time (292 min [114–462] vs. 242 min [122–445], *P* = 0.14; Fig. 1C).

Pelvic lymph node dissection was performed with greater frequency in the culture-guided prophylaxis group compared to the empirical prophylaxis group (93.9% vs. 48.4%, *P* < 0.001). Concomitant urethrectomy was also more prevalent in the culture-guided prophylaxis group (80.3% vs. 62.9%, *P* = 0.03). The estimated blood loss was significantly less in the culture-guided prophylaxis group than in the empirical prophylaxis group (425 mL [80–11,330] vs. 832 mL [95–6979], *P* = 0.02), and the perioperative blood transfusion rate was correspondingly lower (13.6% vs. 59.7%, *P* < 0.001; Fig. 1D).

The 30-day incidence of surgical site infection did not differ significantly between the groups (16.7% vs. 17.7%, *P* = 0.87), including superficial incisional surgical site infection (SSI) (10.6% vs. 9.7%, *P* = 0.86) and organ/space SSI (10.6% vs. 9.7%, *P* = 0.86). However, post-stent removal pyelonephritis occurred significantly less frequently in the culture-guided prophylaxis group compared to the empirical prophylaxis group (3.0% vs. 16.1%, *P* = 0.01).

The incidence of postoperative ileus (15.2% vs. 21.0%, *P* = 0.39) and the reoperation rate (3.0% vs. 8.1%, *P* = 0.20) were not significantly different between the two groups. However, the postoperative transfusion rate was significantly lower in the culture-guided prophylaxis group compared to the empirical prophylaxis group (7.6% vs. 22.6%, *P* = 0.02).

Postoperative complications, as assessed by the Clavien–Dindo classification, showed no significant differences between the groups in the rates of Grades IIIa, IIIb, IVa, or IVb complications.

### Microbiological findings

Table 2 summarizes the microbiological findings of urine cultures obtained before stent removal.

**Table 2:**
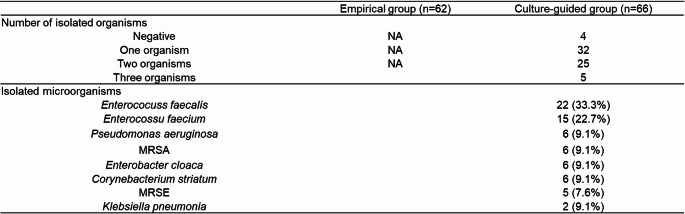
Urine culture results of ileal conduit urine before stent removal

MRSA: Methicillin-resistant *Staphylococcus aureus*, MRSE: Methicillin-resistant *Staphylococcus epidermidis*

*Enterococcus faecalis* was the most frequently isolated organism. Furthermore, *Enterococcus faecium* was frequently isolated, indicating a predominance of *Enterococcus* species. Several antimicrobial-resistant organisms, including *Pseudomonas aeruginosa* and methicillin-resistant *Staphylococcus aureus*, were also commonly detected. In the culture-guided prophylaxis group, prophylactic antibiotics were selected to cover all detected organisms.

### Risk factors for post-stent removal pyelonephritis

Baseline characteristics, stratified by the occurrence of post-stent removal febrile pyelonephritis, are summarized in Table S2. Twelve patients developed febrile pyelonephritis following ureteral stent removal, while 116 patients did not.

The results of the multivariate logistic regression analysis are presented in Table 3. Culture-guided prophylactic antibiotic selection was independently associated with a reduced risk of post-stent removal febrile pyelonephritis (odds ratio, 0.139; *P* = 0.04). Conversely, antithrombotic therapy was identified as an independent risk factor for the development of febrile pyelonephritis.

**Table 3:**
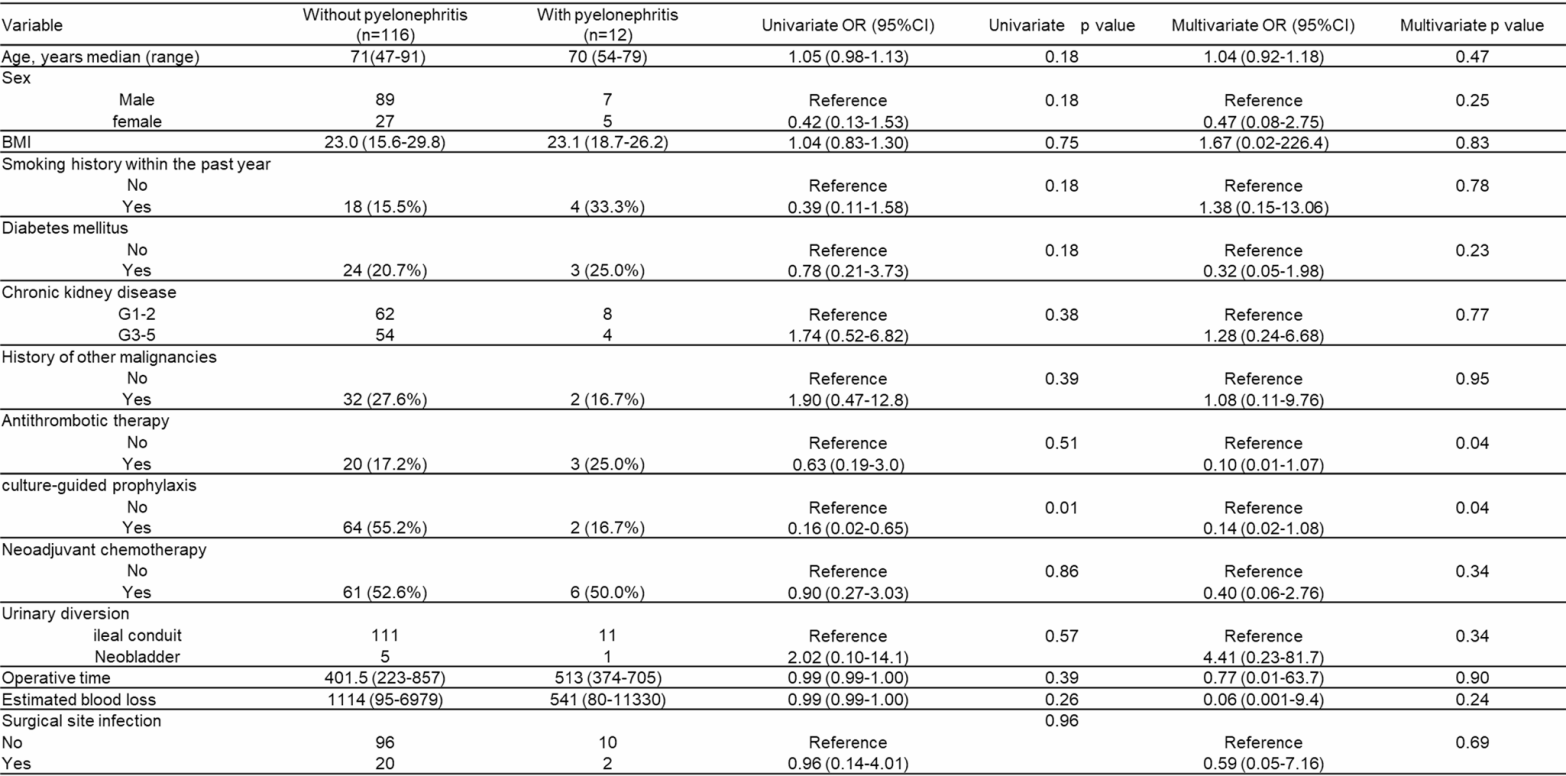
Univariable and multivariable analyses of factors associated with pyelonephritis

CI, confidence interval; OR, odds ratio

### Length of hospital stay and hospitalization costs

**Fig. 2:**
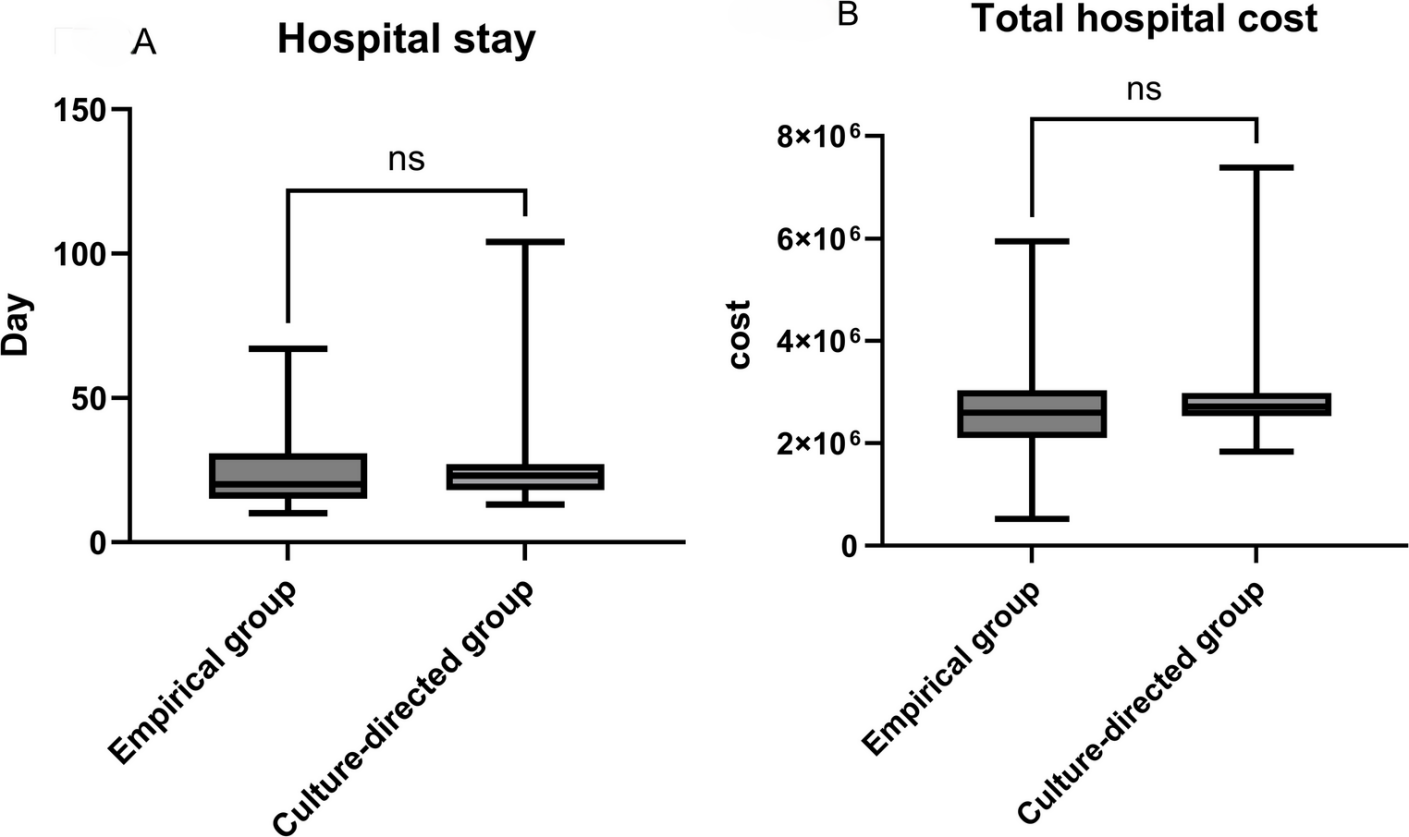
Early postoperative outcomes according to prophylactic strategy. Panels **A** and **B** compare early postoperative outcomes between the empirical prophylaxis and the culture-guided prophylaxis groups. **A** Total length of hospital stay. **B** Total hospital cost

**Fig. 3:**
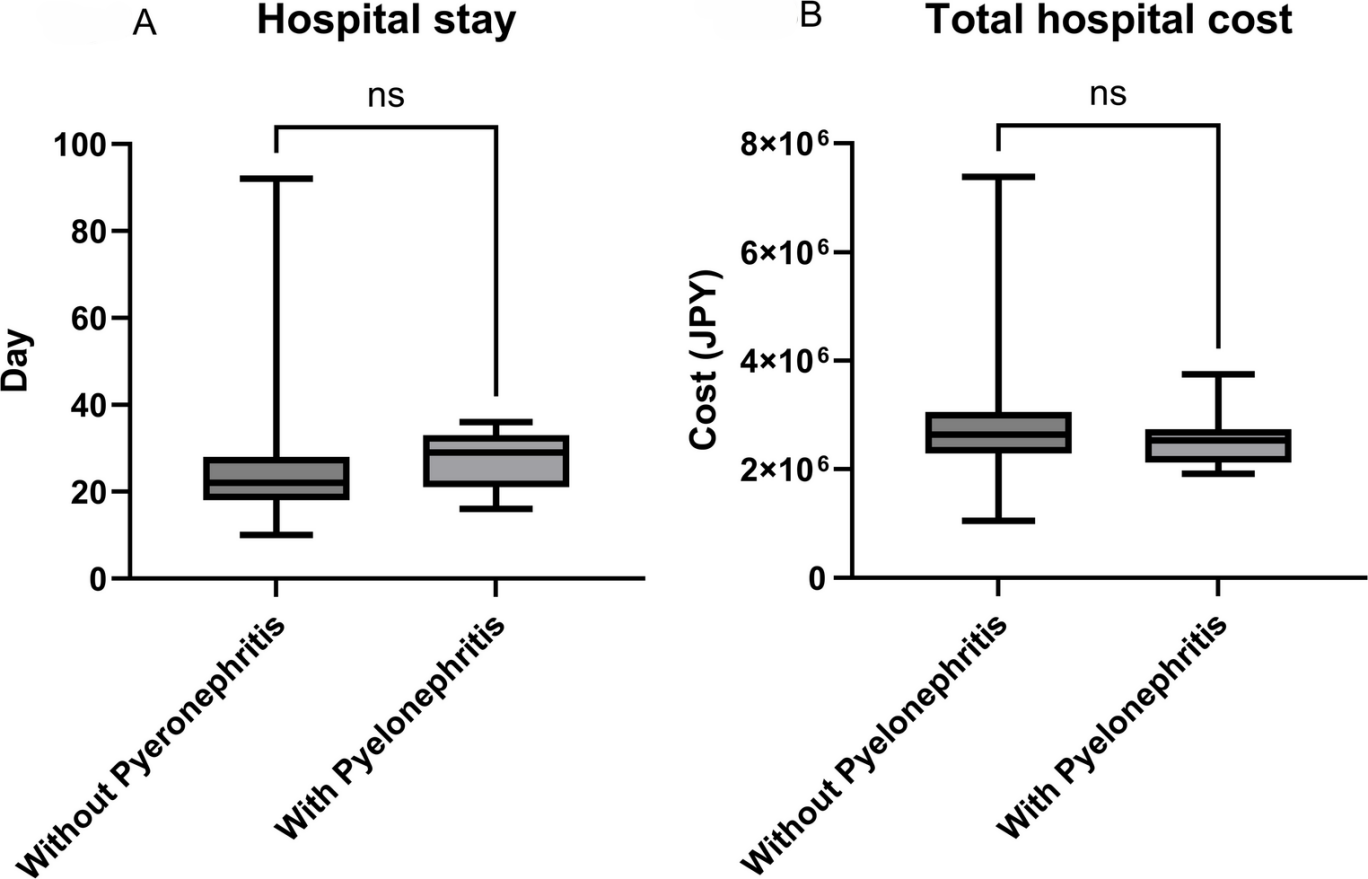
Postoperative outcomes according to the presence of pyelonephritis. Panels **A** and **B** illustrate postoperative outcomes stratified by post-stent removal pyelonephritis. **A** Length of hospital stay. **B** Total hospital cost

The length of hospital stay and total hospital costs (JPY) were compared between the culture-guided and empirical prophylaxis groups (Fig. 2A and B) and between patients with and without post-stent removal febrile pyelonephritis (Fig. 3). No significant differences were observed in the length of hospital stay or total hospital costs between the culture-guided and empirical prophylaxis groups. Similarly, neither the length of hospital stay nor total hospital costs differed significantly between patients who developed febrile pyelonephritis and those who did not.

## Discussion

This study demonstrated that urine culture-guided prophylactic antibiotic selection before ureteral stent removal was associated with a significantly lower risk of febrile pyelonephritis following radical cystectomy with ileal conduit urinary diversion.

Several aspects of surgical practice remain unclear, including the role of perioperative ureteral stenting in patients undergoing radical cystectomy with urinary diversion. Perioperative ureteral stenting is intended to prevent urinary leakage and reduce ureteroenteric anastomotic complications, which account for approximately 30% of postoperative morbidity [2, 11, 12]. However, ureteral stent placement or prolonged stent retention may increase the risk of UTI, potentially necessitating antibiotic treatment or prophylaxis and additional follow-up for stent removal [13].

Despite these concerns, the absence of consensus and high-quality evidence hinders definitive recommendations and the establishment of clear guidelines for interventions aimed at reducing infectious complications following radical cystectomy.

The American Urological Association guidelines on antimicrobial prophylaxis for urological surgery currently recommend a single preoperative dose of cefazolin for cystectomy when the small bowel is used for urinary diversion [14]. While antibiotic prophylaxis is recommended before bladder catheter removal in patients with risk factors, prophylactic antibiotic administration before ureteral stent removal is not recommended [15]. Conversely, the current EAU guidelines do not provide specific recommendations for antibiotic prophylaxis during or after cystectomy or at ureteral stent removal [8].

Furthermore, Parekh et al. [4] reported that the incidence of symptomatic UTIs following radical cystectomy was approximately 26% and 35% after open and robot-assisted surgery, respectively.

In contrast, Beano et al. [16] reported that after obtaining urine cultures from ureteral stents or urostomy bags and administering a single intravenous antibiotic dose tailored to the culture results, the UTI-related readmission rate within 90 days postoperatively increased from 6.6 to 18.6% in patients with negative and positive cultures, respectively. The incidence of urinary tract infections (UTIs) following radical cystectomy remains elevated, and the presence of bacteria within the urinary diversion appears to be a critical factor in postoperative UTI development. Consequently, prophylactic antibiotic strategies targeting these organisms may reduce UTI incidence after radical cystectomy. Nasu et al. [17] evaluated 50 patients who underwent radical cystectomy with intestinal urinary diversion. Among 43 patients for whom antibiotic selection was guided by urine cultures obtained from ureteral stents, only three (7%) developed febrile UTIs after ureteral stent removal, a significantly lower incidence than the 71% observed in patients whose antibiotic therapy was not culture-guided. Wang et al. [13] reported that urine cultures obtained from the ileal conduit on postoperative day 3 and from ureteral stent tips on postoperative day 7, followed by prophylactic antibiotic selection based on these results, reduced febrile UTI incidence from approximately 13.4% to 4.5%. Werntz et al. [18] reported that postoperative prophylactic oral trimethoprim–sulfamethoxazole (160/800 mg daily), nitrofurantoin (100 mg daily), or ciprofloxacin (250 mg daily) reduced the overall postoperative UTI rate from 36% to 12%. Furthermore, prophylactic antibiotic use decreased UTI incidence from 30 to 0% on the day after ureteral stent removal. Shigemura et al. [19] investigated patients who underwent radical cystectomy and received perioperative prophylaxis with tazobactam/piperacillin. Among patients with a positive preoperative urine culture (16 of 49), oral fluoroquinolones were administered 1–2 h before ureteral stent removal. Infectious complications occurred in only one of the 16 patients (6%) who received prophylactic antibiotics. Conversely, UTIs were observed in six of the 33 patients (18%) who did not receive prophylactic antibiotics at stent removal. However, a prospective study by Tobia et al. [20] reported limited concordance between perioperative intestinal or catheter cultures and pathogens identified during subsequent urinary tract infections after cystectomy with intestinal urinary diversion, suggesting that perioperative colonization may not reliably predict later infectious events.

Nevertheless, previous studies suggest that prophylactic antibiotic administration at ureteral stent removal during urinary diversion after radical cystectomy may be associated with a reduced incidence of post-stent removal UTIs. However, prolonged antibiotic exposure is associated with an increased risk of multidrug-resistant organism emergence [21] and *Clostridioides difficile* infections [22]. Therefore, a strategy that targets a limited organism spectrum and uses short-duration prophylactic antibiotic therapy may better balance infection prevention with appropriate antimicrobial stewardship. In this context, our study demonstrated that identifying target organisms and administering narrow-spectrum antibiotics for approximately 2 days can effectively reduce the incidence of UTIs after ureteral stent removal. Moreover, the culture-guided strategy enabled short-course, narrow-spectrum prophylaxis, potentially reducing unnecessary antimicrobial exposure while aligning with current antimicrobial stewardship principles. Notably, the odds ratio for antithrombotic therapy differed between univariable and multivariable analyses. This change likely reflects confounding by perioperative factors included in the adjusted model rather than a direct causal relationship, and therefore should be interpreted with caution given the limited number of events.

Previous studies [5, 18] have reported that common causative pathogens of UTIs after radical cystectomy include *Enterococcus* spp., *Escherichia coli*, and *Staphylococcus aureus*. Moreover, polymicrobial infections have also been reported [23], consistent with our findings. In the present cohort, several frequently isolated organisms, including Enterococcus faecalis, Pseudomonas aeruginosa, Enterobacter cloacae, and Klebsiella pneumoniae, would generally be expected to be covered by fluoroquinolone-based empirical prophylaxis. However, other organisms such as Enterococcus faecium, MRSA, MRSE, and Corynebacterium striatum would not be reliably covered by such regimens and would require glycopeptide-based therapy. These microbiological differences may partly explain the potential benefit of culture-guided prophylaxis. When glycopeptide agents were selected, therapeutic drug monitoring was performed to ensure safety, and in a limited number of cases antibiotics were administered for approximately 72 h.

Total hospital costs tend to be higher for robot-assisted surgery than for open surgery and have been reported to be positively associated with a Charlson Comorbidity Index ≥ 2, the occurrence of complications, and increased length of hospital stay [24].

To the best of our knowledge, no previous study has specifically examined whether pyelonephritis alone affects total hospital costs, as evaluated in the present study. Because pyelonephritis was not associated with increased total hospital costs in our analysis, other postoperative complications may have contributed more substantially to overall hospital costs.

## Limitations

This study has several limitations. First, the retrospective design and single-center setting may limit the generalizability of the findings. Second, the relatively small number of post-stent removal febrile pyelonephritis events may have reduced the statistical power of multivariate analysis and widened confidence intervals, and therefore the results of the multivariable model should be interpreted as exploratory. Third, differences in surgical era and perioperative management may have introduced residual confounding, as robot-assisted surgery was more frequently performed in the culture-guided prophylaxis group. Although multivariate adjustment was performed, unmeasured confounders cannot be fully excluded. In particular, because the introduction of robot-assisted radical cystectomy temporally overlapped with the implementation of culture-guided prophylaxis, the independent effect of antibiotic strategy may not be completely distinguishable from that of surgical approach.　Additionally, urine cultures were obtained from ileal conduit urine rather than directly from the upper urinary tract, which may not fully reflect the microbiological environment at stent removal. Finally, the study focused on short-term infectious outcomes and hospital costs and was not designed to assess long-term outcomes, antimicrobial resistance patterns, or *Clostridioides difficile* infection. Additionally, because ureteral stent removal is performed after ileal conduit creation, urine samples were obtained from an intestinal segment that harbors its own microbiota. Thus, positive cultures from conduit urine may reflect colonization rather than true infection. The relatively low quantitative threshold (≥ 10² CFU/mL) may also have increased detection of non-pathogenic or colonizing organisms. Accordingly, our findings indicate an association between a culture-guided strategy and reduced febrile events, rather than establishing the biological necessity of treating all cultured organisms. Future studies should evaluate whether targeting selected high-risk pathogens or applying higher quantitative thresholds could achieve similar preventive effects while further minimizing antimicrobial exposure.

In conclusion, urine culture–guided prophylactic antibiotic selection before ureteral stent removal after radical cystectomy with urinary diversion was associated with a lower incidence of febrile pyelonephritis in this retrospective cohort. While previous studies have reported that guideline-adherent antibiotic prophylaxis after cystectomy is associated with reduced infectious complications [25], evidence specifically addressing prophylaxis at ureteral stent removal remains limited. These findings should be interpreted cautiously, and further prospective studies are warranted to clarify the clinical impact of this approach.

## Supplementary Information

Below is the link to the electronic supplementary material.

Supplementary Material 1

## Data Availability

The data that support the findings of this study are not publicly available due to ethical and privacy considerations, but are available from the corresponding author upon reasonable request.
