# Supplementary material for: Urine culture–guided antibiotic prophylaxis reduces febrile pyelonephritis after ureteral stent removal following radical cystectomy
Source: World J Urol. 2026 Mar 18;44(1):249. doi: 10.1007/s00345-026-06334-z (PMC12999691; doi:10.1007/s00345-026-06334-z)

Supplement Table 1. Perioperative outcomes (N = 128)


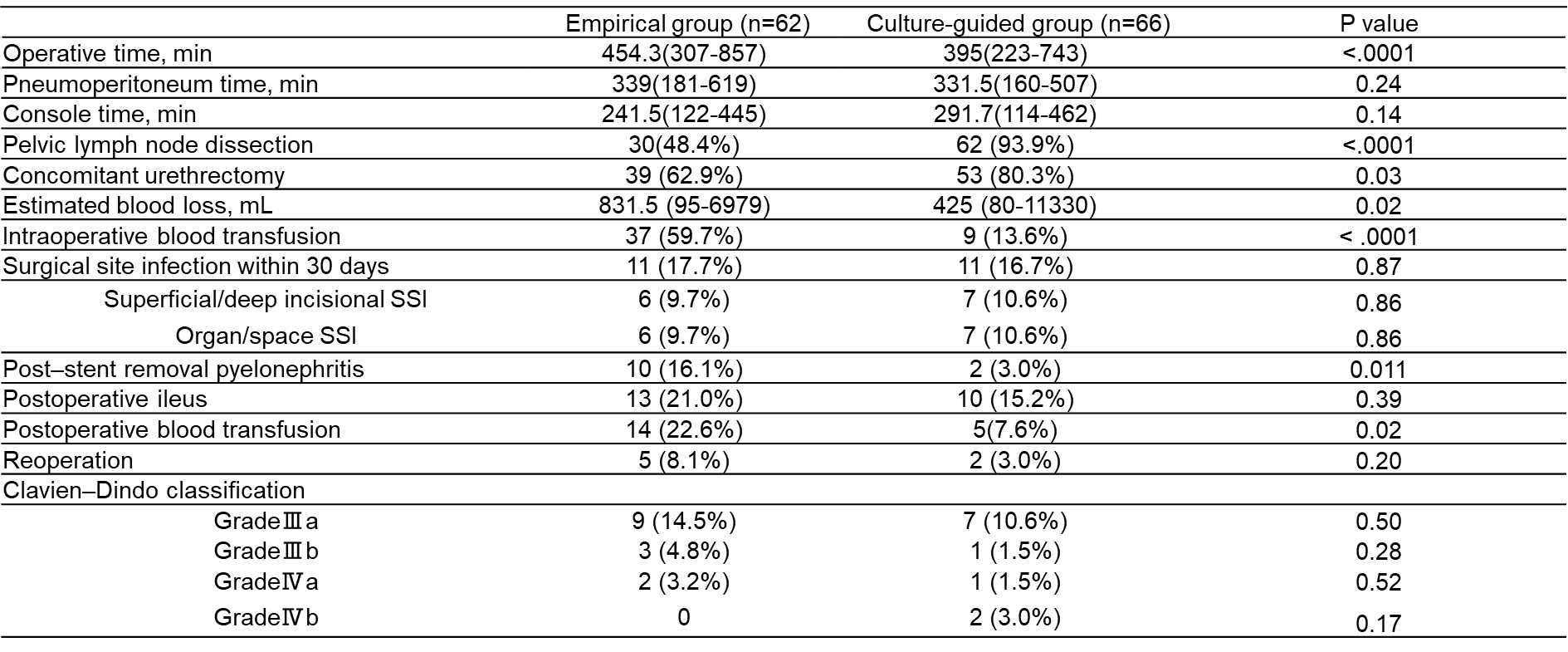


Supplement Table 2. Patient characteristics according to post–stent removal febrile pyelonephritis


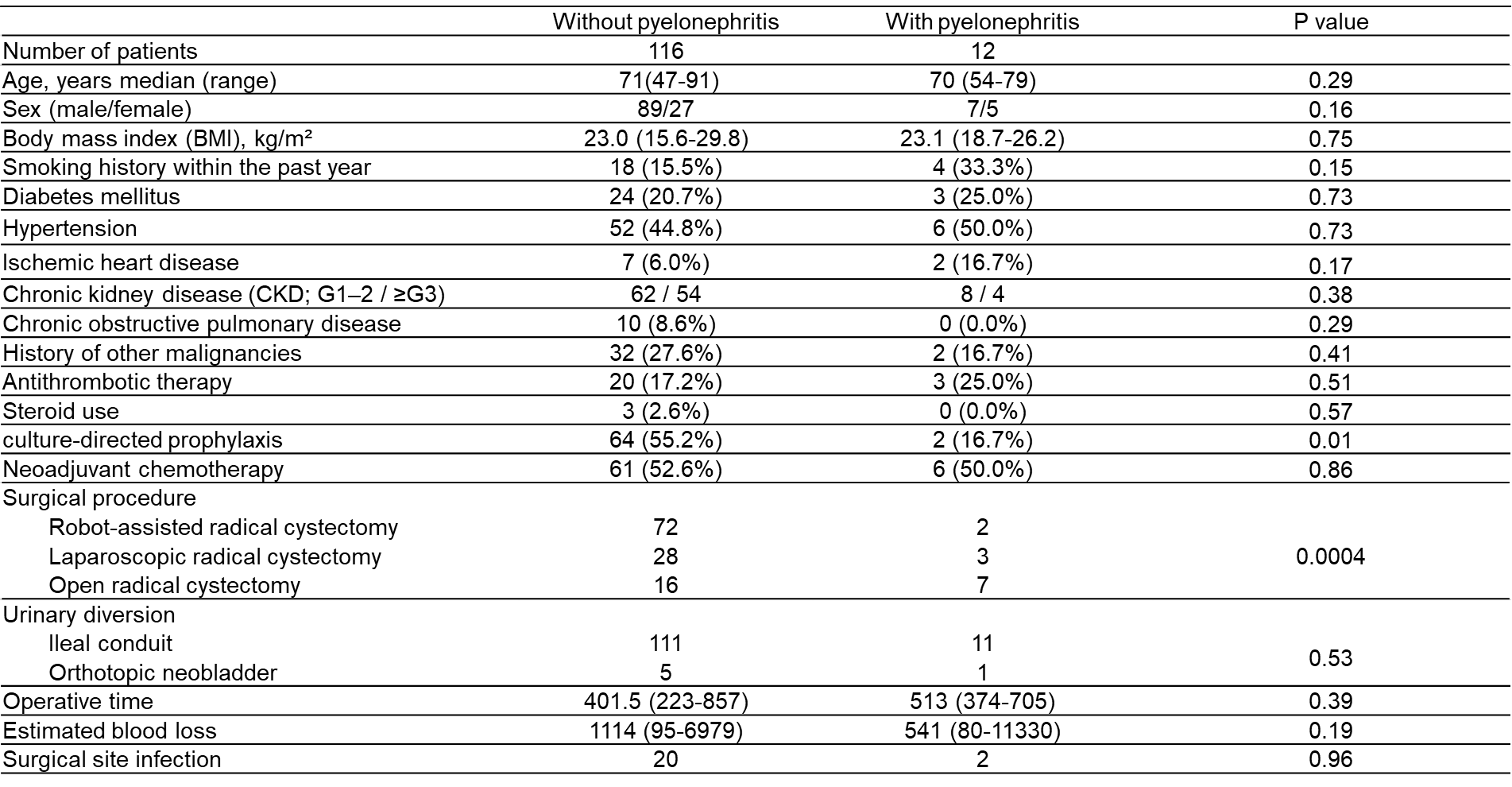

Supplement: Supplementary file 1 — Supplementary Material 1 [file 345_2026_6334_MOESM1_ESM.docx]
